# Elevated ferritin expression in microglia and extracellular amyloid-β deposition are associated with reduced neurofibrillary degeneration in human isocortex, but not allocortex

**DOI:** 10.1177/13872877261470454

**Published:** 2026-07-23

**Authors:** Wolfgang J. Streit, Heidrun Kuhrt, Ingo Bechmann

**Affiliations:** 1 Institute of Anatomy, 9180 Leipzig University, Leipzig, Germany

**Keywords:** Alzheimer's disease, amyloid-β protein, ferritin, grid cells, iron, late-onset Alzheimer's disease, microglia

## Abstract

**Background:**

Prior work in preclinical late-onset Alzheimer's disease (LOAD) focused on neuritic plaque development suggested that intracellular ferritin expression in microglia and extracellular deposition of amyloid-β (Aβ) are innate neuroprotective mechanisms geared specifically towards limiting aging-dependent increases in intracerebral free iron which likely contribute to development of neurofibrillary degeneration (NFD).

**Objective:**

Improve understanding of LOAD pathogenesis.

**Methods:**

Immunohistochemical comparison of the extent of NFD with the intensity of ferritin expression and Aβ deposition in three brain regions, including temporal lobe (entorhinal cortex, hippocampus), frontal, and occipital cortex in 34 non-demented human subjects at Braak stages II-III.

**Results:**

Ferritin-positive microglia are present with similar quantity and intensity in the allo- and isocortices of every individual in the cohort. Extracellular Aβ deposition in the isocortex is observed before substantial NFD develops, but in the allocortex (temporal lobe) there are no Aβ deposits in 50% of subjects despite extensive NFD. Cytoskeletal lesions in the allocortex consist of atrophic grid cells, abundant pretangles, neuropil threads, neurofibrillary tangles, and neuritic plaques; isocortical sites show either no NFD at all or only minimal NFD presenting as solitary pretangles or tangles, neuropil threads, or droplet degeneration spheres from ferroptotic neurons. Presence of degenerating grid neurons in entorhinal cortex coincides with microglial apoptosis.

**Conclusions:**

Neuroprotection *via* ferritin expression and Aβ deposition is more effective in the isocortex than in allocortex. Findings support the hypothesis that degeneration or death of neuroprotective microglia promotes neuronal degeneration.

## Introduction

Neurofibrillary degeneration (NFD), or tau pathology, is known to begin during early life in the locus coeruleus and later spreads to cortical sites, notably the mesial temporal lobe which includes entorhinal cortex and hippocampus.^1–3^ At Braak stages II-III, there is already extensive presence of pretangles, neuropil threads, and neurofibrillary tangles (NFTs) in the entorhinal allocortex, as well as in hippocampal regions, including subiculum, CA1, CA2, and, to a lesser extent, the hilus and basal temporal cortex.^4^ At stages II-III, frontal and occipital isocortices only begin to develop tau pathology and may not show any at all. The gradual progression of tau pathology from allocortex to isocortex may be explained by selective neuronal vulnerability of projection neurons with long and sparsely myelinated axons, yet mechanisms underlying this selectivity have yet to be elucidated.

In the allocortex, substantial tau pathology up to stage III can develop in the absence of amyloid-β (Aβ) deposition, which stands in stark contrast to isocortex where Aβ deposits are generally detected before significant tau pathology is seen. While presence of Aβ in the isocortex prior to the onset of tau pathology has been an important argument in support of the Aβ hypothesis,^5^ absence of Aβ in the allocortex during NFD development represents a conundrum and contradicts the Aβ theory. Efforts to explain this mystery include claims that soluble, yet invisible toxic Aβ oligomers are the primary toxic agent,^6,7^ as well as introduction of primary age-related tauopathy as a distinct condition thought by some to be different from preclinical late-onset Alzheimer's disease (LOAD).^8–10^ Despite such efforts LOAD pathogenesis remains uncertain and controversial.

In the current paper, our intention is to investigate LOAD pathogenesis from a different perspective, namely, that Aβ plays a role that differs from the traditional view of Aβ as a neurotoxic agent triggering NFD. Our view of Aβ deposition as a neuroprotective response stems from prior work focused on neuritic plaque formation where we found that Aβ is deposited very precisely on droplet degeneration spheres that arise from ferroptotic neurons fragmenting into phospho-tau (p-tau)-positive droplets and dystrophic neurites.^11^ Aβ deposition occurring after neuronal ferroptosis then results in the formation of neuritic plaques. It is conceivable that such targeted Aβ deposition helps contain iron leakage after ferroptosis *via* chelation acting as an endogenous mechanism for regulating intracerebral iron content.^12^

Elevated intracerebral iron (iron dyshomeostasis) has been implicated in the development of aging-related neurodegenerative diseases, including AD.^13–19^ Work from this laboratory has offered insights into the relationships between dysregulated brain iron content and the classical lesions that define AD, plaques and tangles.^20^ Specifically, we have identified iron-mediated cell death (ferroptosis) as a primary event in the formation of neuritic plaques. Neuronal ferroptosis presents as droplet degeneration, which is accompanied by accumulation of ferritin-positive microglia sequestering iron. Ferroptotic droplets and cell fragments are subsequently encased by Aβ, an additional mechanism for containing excessive amounts of free iron. We have proposed that expression of the iron storage protein, ferritin, in healthy microglial cells, as well as the deposition of Aβ represent distinct endogenous protective mechanisms for countering/limiting aging-related increases in brain iron and thus aiding in the prevention/slowing of further NFD. We believe that expression of ferritin in microglial cells functions constitutively to regulate excess extracellular iron through uptake and sequestration within the microglial cytoplasm. Aβ deposition, which occurs later than ferritin expression, chelates iron extracellularly; it accumulates rapidly in nascent neuritic plaques to contain excess iron released from ferroptotic neurons.^11^ To further investigate these protective responses, in the current study we have examined and compared the occurrence of microglial ferritin expression and Aβ deposition in relation to the development of NFD in the allo- and isocortex.

Differential neuronal vulnerability decreases with increasing cortical differentiation from allo- to isocortex,^21^ and phylogenetically older neurons are more vulnerable to degeneration. The current study shows that neurons in allocortex are undergoing NFD despite protective ferritin expression supporting the thought that allocortical cells are highly susceptible to degeneration. The same may apply also to dopaminergic cells in substantia nigra which are prone to degenerate even though they contain iron-binding neuromelanin and are often surrounded by numerous ferritin-positive microglia.^22,23^ Thus, microglia-mediated neuroprotection *via* iron sequestration may be limited in terms of reducing neuronal vulnerability. Conversely, degeneration of neuroprotective microglia due to a variety of reasons, including prolonged ferritin expression and ensuing senescence, likely increases neuronal vulnerability.^24–27^ Microglial degeneration and senescence are the principal concepts underlying the microglial dysfunction hypothesis,^28–30^ which has guided our prior work in LOAD pathogenesis. The idea receives further support also in the current study.

## Methods

### Subjects and case selection

Case selection was performed from a pool of individuals who came to autopsy in 2014–2023 as a matter of routine procedure following their deaths at the Leipzig University Hospital. In their individual contracts that govern medical treatment, upon admission all patients provided written consent to the scientific use of tissue removed and stored after any biopsy or during autopsy. A procedural criterion used was a postmortem interval of less than 48 h. Following removal, whole brains were fixed by immersion in 4% buffered formaldehyde for at least 2 weeks. Brains were released by the Department of Neuropathology after complete post-mortem evaluation by clinical neuropathologists. The medial temporal lobe, frontal and occipital poles, were dissected from 2 cm thick coronal slices and then embedded in polyethylene glycol, followed by sectioning at 100 µm on a macrotome, as described previously.^11,24^ Thirty-three out of 34 cases listed in Table 1 were selected based on being staged at Braak II-III or lower, during neuropathological evaluation. A 34^th^ case at Braak stage IV was included for comparison as it revealed maximal ferritin and amyloid expression, and also significant tau pathology in both allo- and isocortex. In this way, we assembled a cohort of individuals who were likely to have been in a stage of LOAD that was within the transitional period from non-symptomatic to symptomatic disease. The cases in Table 1 are sorted according to increasing age. All cases were individuals whose medical history, including clinical neuropathology evaluation, did not include mention of clinical dementia; they were thus considered non-demented subjects.

**Table 1.** Patient data.

Case ID	Age/Sex	Braak stage	Ferritin EC	Ferritin FC/OC	Aβ EC*	Aβ FC/OC	AT8 EC	AT8 FC/OC	TUNEL
1	54/m	II-III	++	++	A0	intraparenchymal, diffuse	preT, NPTs, NFTs	none	ND
2	58/m	II-III	±	+	A0	intraparenchymal, diffuse and perivascular	preT, NPTs, NFTs	none	Infrequent
3	63/m	II-III	++	+	A2	perivascular only	preT, NPTs, NFTs	none	ND
4	63m	II-III	±	+	A0	none	preT, NPTs, NFTs, DD	few preT, NPTs	Infrequent
5	66/f	I-II	++	++	A1	frequent intraparenchymal	preT, NPTs, NFTs	none	ND
6	66/f	II-III	±	+	A1	intraparenchymal, diffuse	preT, NPTs, NFTs	few NPTs	ND
7	68w	II-III	+	+	A0	frequent intraparenchymal	preT, NPTs, NFTs	none	ND
8	68/w	II-III	±	+	A0	intraparenchymal	preT, NPTs, NFTs	none	Frequent
9	69/m	II	++	++	A0	none	preT, NPTs, NFTs	none	ND
10	70/m	II-III	+++	+++	A1	frequent intraparenchymal	preT, NPTs, NFTs, DD, NPs	few preT, NPTs, DD	ND
11	70/f	II-III	±	++	A2	frequent intraparenchymal, diffuse	preT, NPTs, NFTs, DD	preT, NPTs, DD	ND
12	71/f	III	+++	+++	A2	frequent intraparenchymal	preT, NPTs, NFTs, DD, NPs	NPTs, DD, NPs	ND
13	71/f	II-III	+++	+++	A2	intraparenchymal, diffuse	preTs, NPTs, NFTs, DD	few preT, NPTs, DD	ND
14	72/m	II-III	+	+	A0	none	preT, NPTs, NFTs	none	ND
15	73/f	II	+	+	A0	intraparenchymal	preT, NPTs, NFTs	none	ND
16	74/m	I-II	+++	++	A1	intraparenchymal	preT, NPTs, NFTs	none	ND
17	74/f	II-III	++	++	A1	perivascular, intraparenchymal	preT, NPTs, NFTs, DD	few preT, DD	ND
18	74/m	II-III	±	+	A0	none	preT, NPTs, NFTs	none	ND
19	74/f	II-III	++	+	A0	perivascular, intraparenchymal	preT, NPTs, NFTs	none	ND
20	75/m	II-III	+	++	A0	Intraparenchymal, diffuse	preT, NPTs, NFTs	few preT, NPTs	ND
21	80/f	II-III	++	++	A0	none	preT, NPTs, NFTs	few preT	ND
22	80/m	II-III	++	+	A2	frequent intraparenchymal	preT, NPTs, NFTs, DD, NPs	few preT	Infrequent
23	80/m	II-III	++	+	A0	none	preT, NPTs, NFTs	few preT, NPTs, NFTs	ND
24	81/f	II-III	++	++	A1	none	preT, NPTs, NFTs	few preT, NPTs	ND
25	81/f	II-III	+	++	A0	none	preT, NPTs, NFTs, DD, NPs	few preT, NPTs	ND
26	83/m	II-III	++	+	A0	perivascular	preT, NPTs, NFTs	few preT, NPTs	ND
27	85/m	II	++	+ perivascular	A1	perivascular	preT, NPTs, NFTs, DD	none	ND
28	86/f	II-III	±	++	A0	none	preT, NPTs, NFTs	few preT, NPTs	ND
29	89/m	II-III	++	+	A0	intraparenchymal	preT, NPTs, NFTs	preT, NPTs, DD	ND
30	89/f	II-III	++	++	A2	ND	preT, NPTs, NFTs, DD	few preT	ND
31	89/m	II-III	+	+	A2	frequent intraparenchymal	preT, NPTs, NFTs, DD, NPs	preT, NPTs, NFTs, DD	Infrequent
32	9	II-III	+++	+++	A2	intraparenchymal	preT, NPTs, NFTs, DD, NPs	none	ND
33	91/f	II-III	+	+	A2	intraparenchymal	preT, NPTs, NFTs, DD, NPs	none	ND
34	93/m	IV	+++	+++	A2	frequent intraparenchymal	preT, NPTs, NFTs, DD NPs	DD, NPs	Infrequent

Ferritin staining score: ± occasional (rare) cells; + moderate numbers of cells; ++ widespread immunoreactivity; +++ widespread, strong immunoreactivity and/or clusters.

*Aβ staging according to Thal et al. (2002).^32^

Aβ deposition in FC and OC is often similar; when not, max. occurrence in either region is listed.

preT: pretangles; NPTs: neuropil threads; NFTs: neurofibrillary tangles; DD: droplet degeneration (ferroptosis); NPs: neuritic plaques; ND: not determined.

### Immunohistochemistry

Immunolabeling for the iron storage protein, ferritin, was performed using anti-ferritin polyclonal antibody (rabbit anti-horse spleen ferritin, Sigma, F6136, diluted at 1:800). A monoclonal antibody against human PHF-tau, clone AT8 (mouse monoclonal, 1:2000, Thermo Fisher Scientific, MN1020, diluted at 1:500), was used for detecting structures containing p-tau, including neuropil threads, pretangles, NFTs, and neural components of neuritic plaques. Aβ was detected using a monoclonal mouse antibody (clone NAB 228, Sigma, A8354, diluted at 1:5000).

For immunostaining using peroxidase-based procedures, sections were incubated free-floating in blocking buffer containing 10% goat serum, 0.1% Triton-X 100 in PBS for 2 h before applying the primary antibodies. Primary antibodies, diluted in PBS containing 1% goat serum and 0.3% Triton in PBS, were applied to sections and incubated overnight at 4˚C. After several washes, sections were incubated with either a biotinylated goat anti-mouse IgG (Sigma, B7264) or a biotinylated goat anti-rabbit IgG secondary antibody (Vector, BA-1000), diluted 1:200 in PBS-T supplemented with 2% normal human serum, for 2 h at room temperature. Sections were then incubated with Extravidin conjugated to horseradish peroxidase (Sigma E2886) and visualized using DAB H_2_O_2_ substrate generating a brown reaction product (Sigma 3,3’-diaminobenzidine tablets, Sigma, D-5905). Selected sections were counterstained with hematoxylin after immunostaining. Negative controls consisted of omitting either the primary antibodies or incubating primary antibodies with mismatched secondary antibodies (e.g., primary mouse with biotinylated goat-anti-rabbit). Double labelling for ferritin/AT8 was performed sequentially by completing the substrate reaction (DAB for brown color) with the first primary antibody, followed by two brief (10 min) washes before application of the second primary antibody ending with Vector SG substrate for a black reaction product (Cat. No. SK-4700). Selected sections were counterstained with hematoxylin. All slides were dehydrated through ascending alcohols, cleared in xylene and coverslipped with Canada Balsam. Preparations were examined blindly (knowing only age, sex, and case number) and photographed using a Zeiss photomicroscope. All cases studied were staged for NFD,^2,31^ and for phases of Aβ deposition,^32^ and received an ABC score. Scoring for ferritin was as described in a prior study,^33^ and as noted in the footnotes of Table 1.

### TUNEL assay

Apoptosis of microglia was evaluated using ApopTag^®^ Peroxidase *In Situ* Apoptosis Detection Kit (Millipore Corporation S7100). The procedure was carried out according to the manufacturer's instructions in paraffin sections of mesial temporal allocortex from six individuals, as shown in Table 1. Since spatial and temporal occurrence of apoptotic microglia in non-demented humans is likely to be low,^34^ six individuals were chosen based primarily on age distribution, that is, we wanted to evaluate microglial apoptosis across the age range in our cohort (from 58 to 93 years-old) to determine if aging affects microglial apoptosis. Slides were evaluated qualitatively by enumerating labeled cells with typical microglial morphology. Previous work with a similar kit in rats had shown that apoptotic microglia can be identified by positive staining of cytoplasmic processes.^35^

## Results

**Figure 1.**
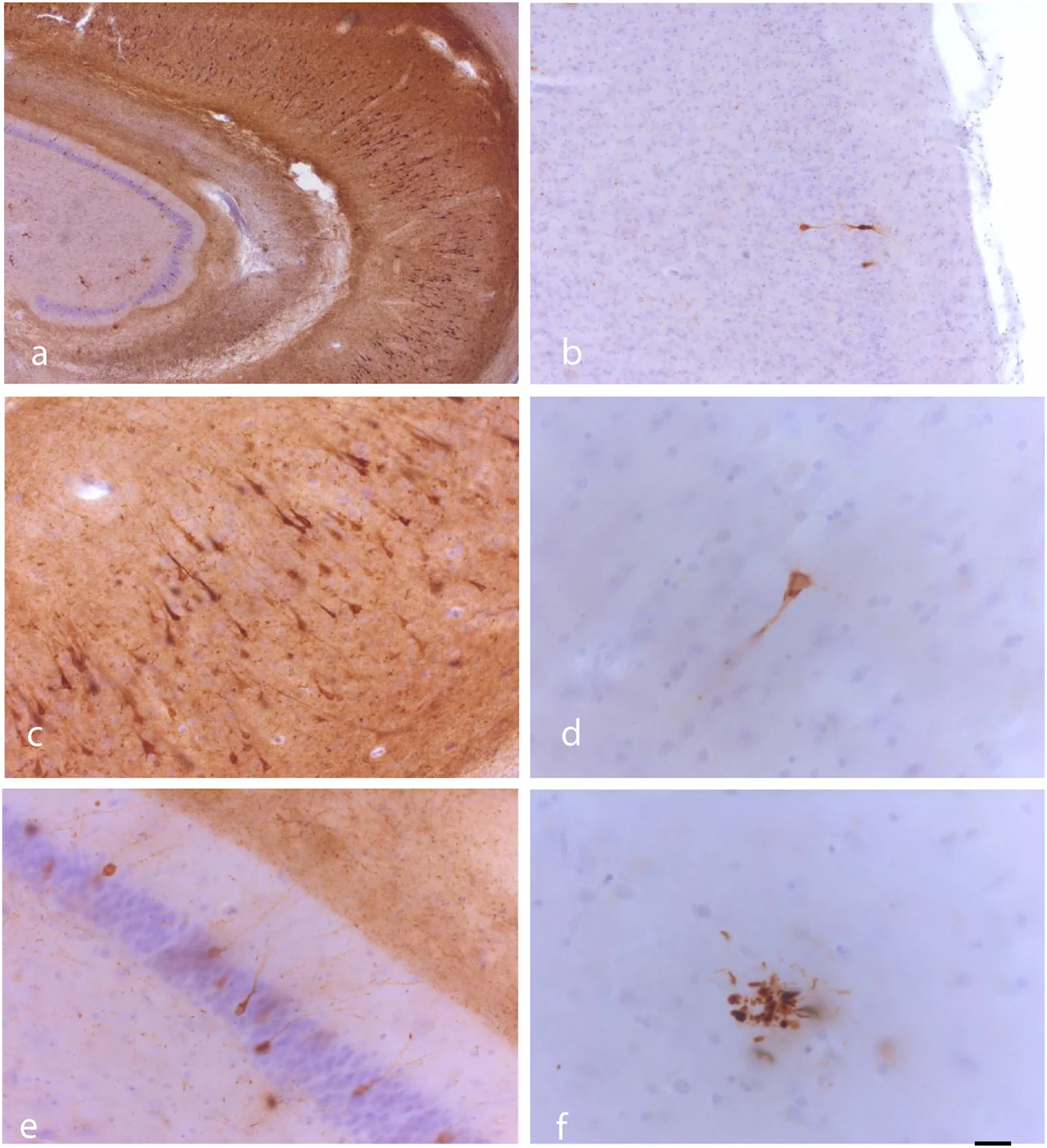
Neurofibrillary degeneration is much greater in allocortex than isocortex; AT8 immunohistochemistry. a, c, e, images of the hippocampus from 80-year-old female. Note the great density of phospho-tau positive neurites and neuronal somata (pretangles and tangles) in Ammon's horn (a, c), and to a lesser extent in the dentate hilus, where individual tau-positive granule cells can be distinguished (e). b, d, f show minimal tau pathology in the temporal cortex (b) of a 74-year-old male, and in the frontal cortex of an 89-year-old female (d,f). Isolated pretangles (b,d), as well as a droplet degeneration sphere (f) are evident. Scale bar: a 800 µm; b,c 200 µm; e 100 µm; d,f 50 µm.

Table 1 summarizes all findings from the current study of 34 individuals of both sexes. All cases are listed in Table 1 according to ascending age, spanning a range from 54 to 93 years old. The results in Table 1 show semi-quantitative scores of ferritin-positive microglia in the allocortical entorhinal region (EC), including hippocampus, and in the frontal cortex (FC) and occipital cortex (OC). Measures in the FC and OC collapse into one column because within the same individual both regions consistently show very similar results. The same is true for Aβ deposition in FC and OC, regions that present matching features of Aβ within the same subject characterized as intraparenchymal or perivascular deposits of diffuse Aβ. Nine out of 34 individuals show no Aβ deposits at all in FC and OC. Aβ deposition in the EC is scored using Thal's staging system; 17 out of 34 individuals showed no Aβ deposits at all (A0) in the EC sections. Table 1 also summarizes neurofibrillary lesions in two columns labeled AT8 EC and AT8 FC/OC. Again, there is great similarity in extent of neurofibrillary lesions between FC and OC, hence both are collapsed into one column. All cases show presence of NFD in the EC, where it is often substantial, as shown in Figure 1. In contrast, in the FC/OC nearly one half of cases (15/34) showed no NFD at all, and all others revealed only minimal lesions consisting of isolated pretangles, neuropil threads, or droplet degeneration spheres (Figure 1). The last column in Table 1 shows findings regarding TUNEL histochemistry in 6 individuals. Five out of the 6 subjects studied do not show more than one or two labelled microglia per section. However, one 68-year-old female revealed an unusually large accumulation of TUNEL-positive microglia in the entorhinal cortex that was coincident with extensive NFD of grid neurons (see below).

**Figure 2.**
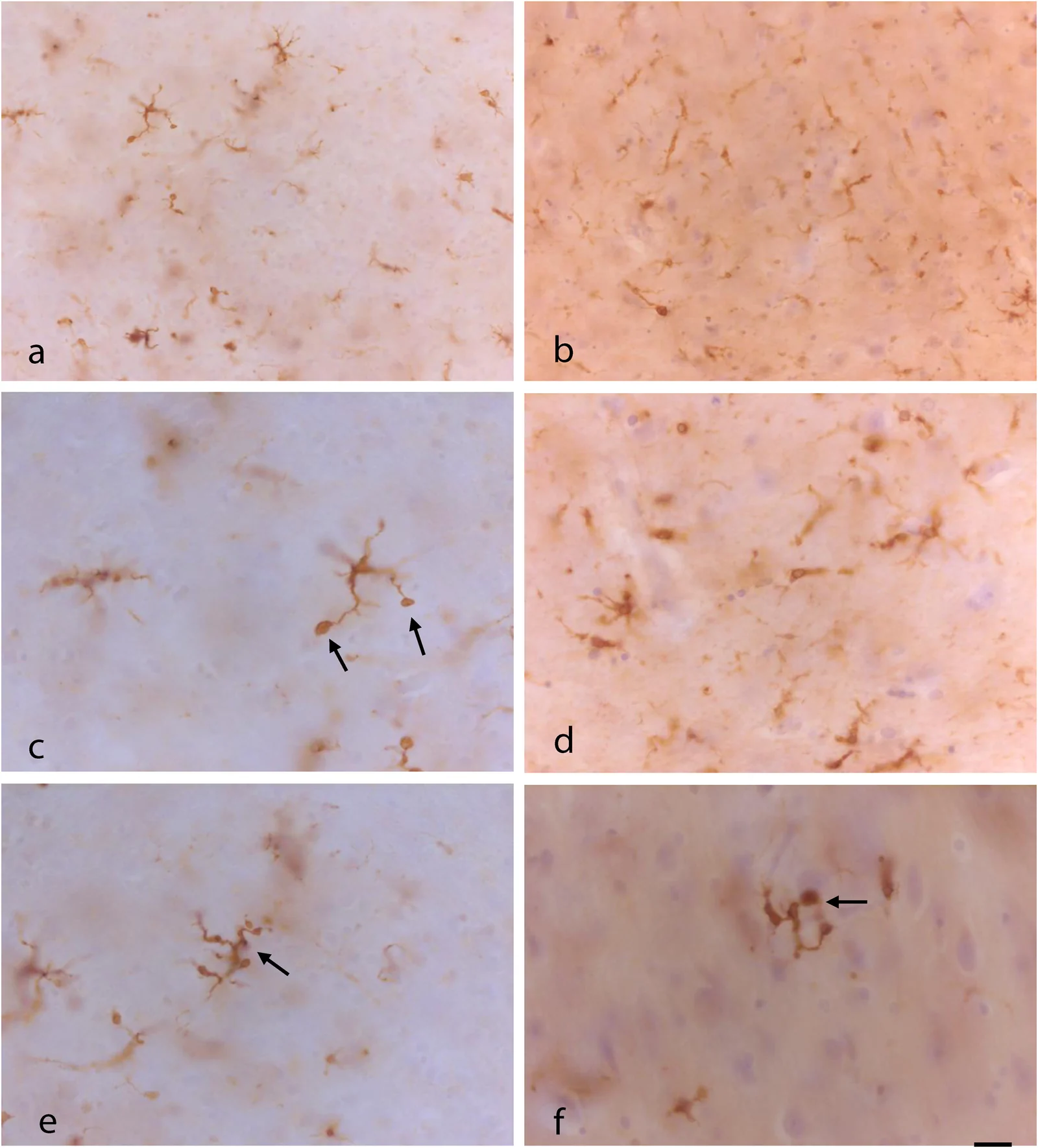
Ferritin expression in microglia is similar in allo- and isocortex. a, c, e, images from occipital isocortex show ferritin-positive microglia which are mostly ramified and slightly dystrophic. Dystrophy presents as spheroidal swellings (arrows in c,e,f). b, d, f, images from entorhinal allocortex also show ferritin-positive microglia which are mostly ramified and slightly dystrophic. All images are from one 84-year-old female. Scale bar: a,b 100 µm; c-f 50 µm.

As shown in Table 1 and in Figures 1 and 2, it is evident that NFD in the allocortex is much greater than in the isocortex, yet microglial ferritin expression is very similar in both allo- and isocortex in any given individual. While all 34 cases in this study show presence of ferritin-positive microglia, there is variation between individuals in terms of the number of ferritin-positive microglia (Table 1). Often, the ferritin score is quite low in individuals who also score A0 in the entorhinal region. The morphology of individual ferritin-positive microglial cells is mostly ramified, although some exhibit clear signs of dystrophy, such as spheroidal swellings located at the end of cytoplasmic processes (Figure 2). With aging, there is a tendency towards increasing ferritin expression and increasing dystrophy, suggesting that dystrophy and ferritin expression are linked.^25,33,36^ Activated (hypertrophic) microglia and clusters of ferritin-positive microglia are not prevalent in the current cohort, which may be related to the fact that cases were at relatively low Braak stages. The fact that all 34 individuals show ferritin expression while many do not show Aβ deposits supports the idea that ferritin expression is the primary iron containment mechanism and triggered early during preclinical LOAD.

**Figure 3.**
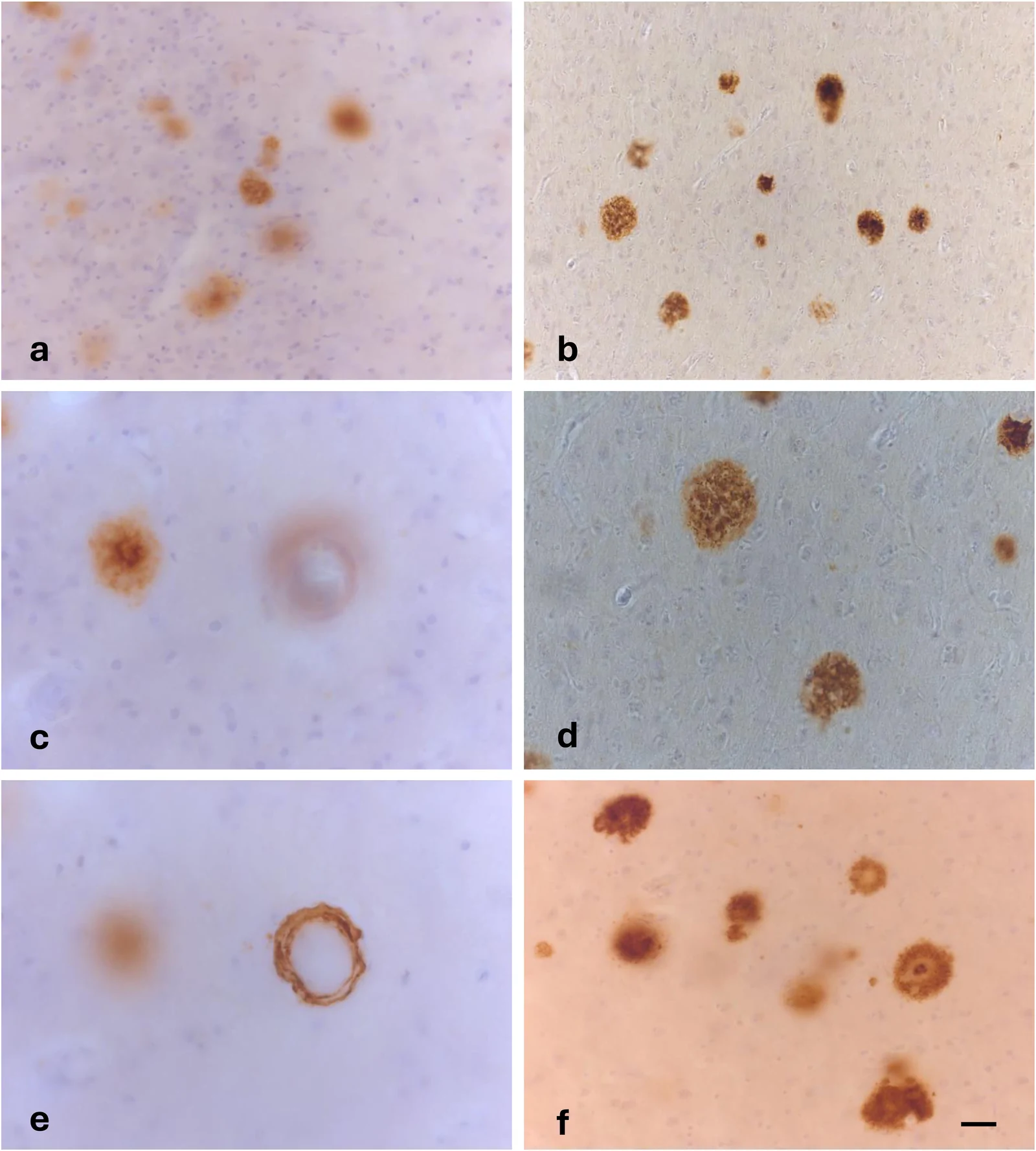
Deposition of Aβ occurs in both allo- and isocortex. a, c, e, images from occipital isocortex show intraparenchymal and perivascular Aβ deposits from two individuals: a 71-year-old female (a), and an 89-year-old female (c, e). Panels c and e display the same microscopic field in different focal planes to show an intraparenchymal deposit next to a perivascular one. b, d, f, images from the entorhinal allocortex show intraparenchymal diffuse deposits or varying sizes from one 71-year-old female. There are only few dense core deposits. Scale bar: a, d, f 100 µm; b 200 µm; c, e 50 µm.

**Figure 4.**
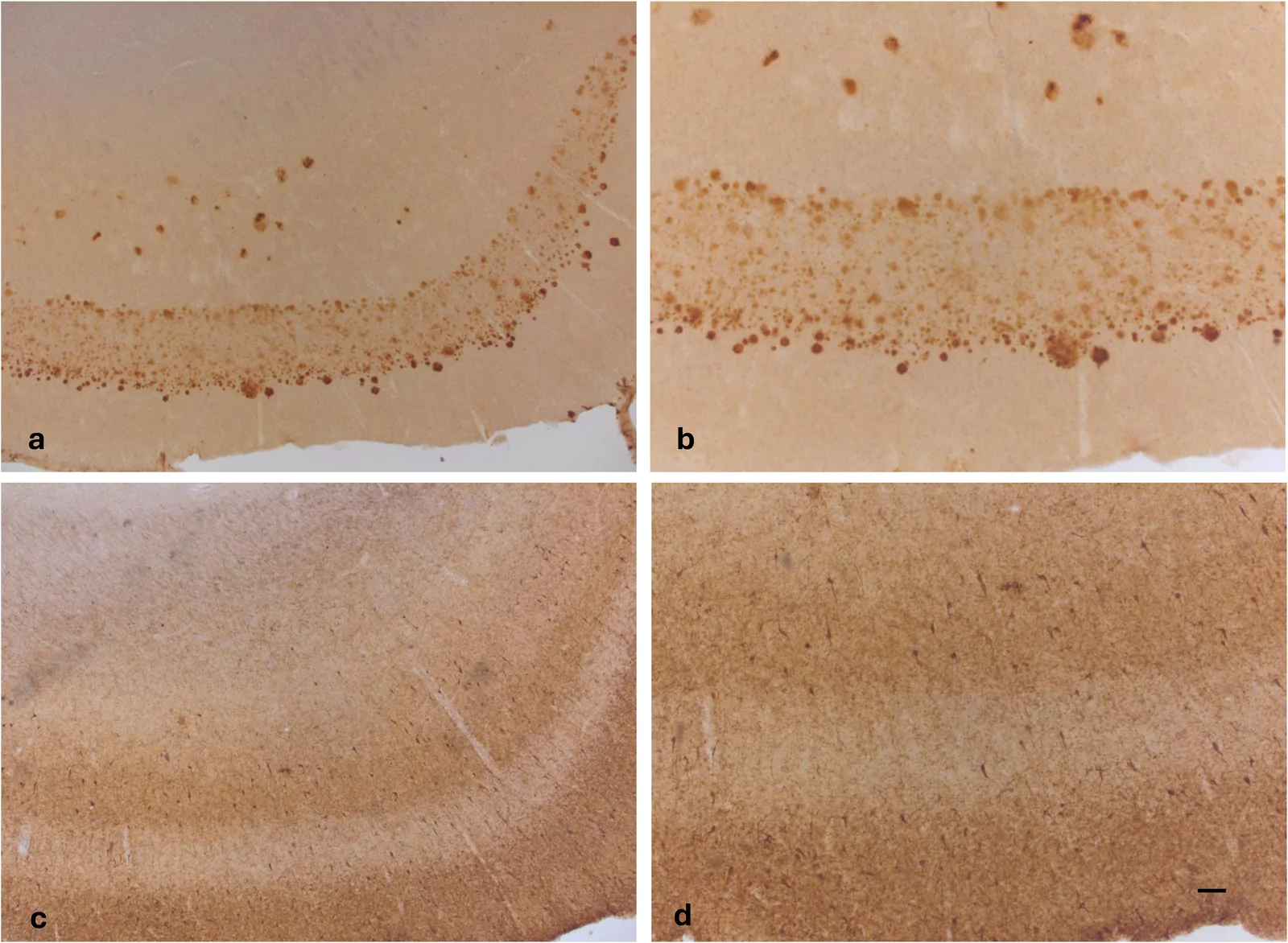
NFD is reduced in an area of intense Aβ deposition in the entorhinal cortex. a, b, Aβ immunostaining; c, d, AT8 immunostaining. A dense band of Aβ deposits, probably in layers pre-β and pre-γ, is shown at two different magnifications (a,b). Adjacent sections (c,d) show reduced staining intensity for phospho-tau in the same band. 80-year-old male. Scale bar: a,c 500 µm; b,d 250 µm.

Deposition of Aβ is quite variable, and seemingly haphazard in our cohort. Absence/presence of Aβ in the allocortex does not predict absence/presence of deposits in the isocortex and *vice versa*. Most Aβ deposits are diffuse in nature with considerable size variations; only few are mature dense core deposits. The deposits occur mostly intraparenchymally but in some cases also perivascularly (Figure 3, Table 1). A protective effect of Aβ in terms of reducing NFD is supported by the finding in Figure 4 showing attenuation of NFD in an entorhinal area containing abundant Aβ deposits.

**Figure 5.**
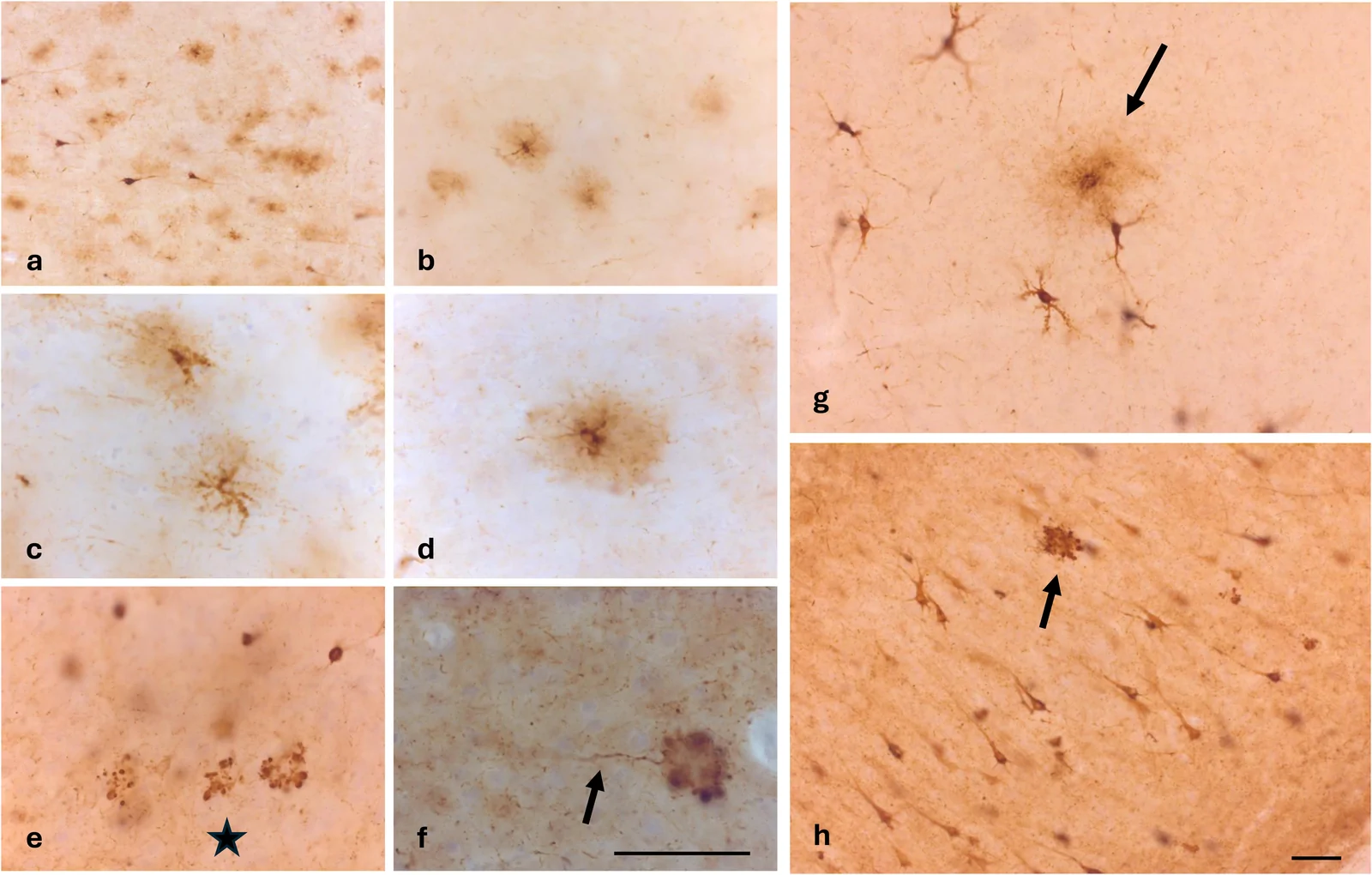
Atrophic NFD of grid neurons is different from droplet degeneration of pyramidal cells; AT8 staining. a-d, images of p-tau-positive grid neurons in the entorhinal cortex from a 68-year-old female. These stellate neurons, arranged in hexagonal arrays (a), reveal atrophy of their distal dendritic tree giving them a fried egg appearance. Remnants of cell bodies remain visible in the center (b-d). e, f, shows examples of droplet degeneration where neuronal somata and processes disintegrate into droplet spheres, three of which are shown in bottom half of panel e (asterisk) opposite of three pretangles in upper half; in panel f, the arrow points to an axon extending from the degenerated cell body; 71-year-old female. g, h, show atrophic degeneration in the dentate hilus (arrow in g), and a large droplet degeneration sphere intermingled with numerous pretangles in Ammon's horn (arrow in h); 80-year-old male. Scale bar a-f: 1000 µm (a), 500 µm (b, e, f), 250 µm (c, d). Scale bar g-h: 250 µm.

During evaluation, one case of a 68-year-old female individual (case 8, Table 1) stood out, showing numerous p-tau-positive neurons in the entorhinal cortex arranged in a hexagonal pattern and each neuron displaying a fried egg appearance (Figure 5a-d). This type of tau pathology has not been reported, and it differs from the common pretangles, tangles, neuropil threads, and droplet degeneration spheres that are also present in this individual. We have determined that the p-tau-positive cells in the medial entorhinal cortex are grid neurons, cells responsible for spatial orientation that were first discovered in 2005.^37^ Apparently, the fried egg appearance of these degenerating grid cells derives from their stellate morphology where distal dendrites disappear or appear fuzzy, likely due to retrograde degeneration (or atrophy) accompanying ongoing tau pathology. Such atrophic NFD of grid cells is different from droplet degeneration of AT8-positive cortical pyramidal cells and mossy neurons in the dentate hilus identified in our prior work.^11^ As shown in Figure 5a-f, the two types of degenerating neurons can be distinguished based on their morphology as seen in AT8-stained preparations. Pyramidal and mossy neurons undergo droplet degeneration (ferroptosis) where somata and processes break up into multiple p-tau-positive droplets of different sizes (Figure 5e, f, h). In contrast, grid neurons undergoing atrophic degeneration seem to be experiencing a retrograde atrophic process where neurites are dying back and produce the distinct fried egg morphological presentation (Figure 5a-d, g). Table 2 summarizes and contrasts features of droplet degeneration and atrophic degeneration. It is of interest within the current context of iron containment mechanisms that this individual (case 8) also shows low or minimal ferritin expression, as well as a lack of Aβ, suggesting that protective mechanisms were not triggered or triggered insufficiently. Atrophic NFD of stellate neurons is also observed in the dentate hilus (Figure 5g).

**Table 2.** Atrophic degeneration versus droplet degeneration of neurons.

Atrophic degeneration	Droplet degeneration
fried egg appearance	appears as droplet degeneration spheres
dying back of processes	breaking up of soma and processes
affects single neurons	can affect single neurons or multiple neighboring neurons
not derived from pretangles	derived from pretangles
not accompanied by Aβ deposition	accompanied by Aβ deposition
accompanied by minimal ferritin expression	accompanied by intense ferritin expression
accompanied by microglial apoptosis	not accompanied by microglial apoptosis
accompanied by extreme microglial dystrophy	accompanied by modest microglial dystrophy
secondary to microglial apoptosis	secondary to neuronal ferroptosis

**Figure 6.**
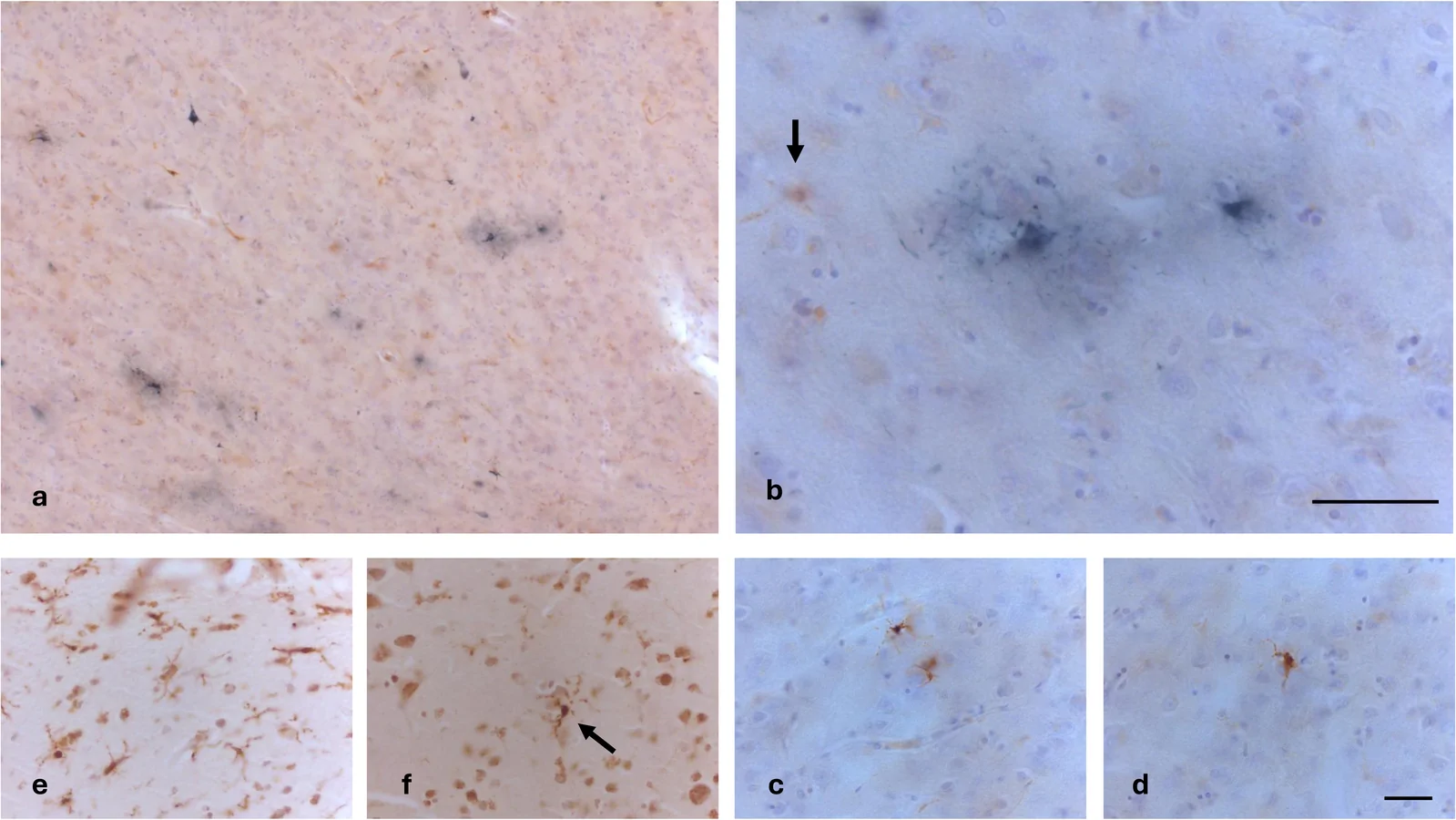
Atrophic NFD of grid neurons is accompanied by weak ferritin expression, microglial dystrophy and microglial apoptosis. a-d, entorhinal cortex double stained with AT8 (black) and ferritin (brown). Low power view (a) shows weak staining of ferritin-positive microglia surrounding degenerating neurons (black). High power view shows a faintly ferritin-positive microglial cell (arrow in b). c,d, ferritin-positive dystrophic microglia with stunted processes. e,f, TUNEL staining. Numerous ramified microglia are shown in e. Single dystrophic microglial cell with fragmented processes in f (arrow). Scale bar a-b: 500 µm (a), 125 µm (b). Scale bar c-f: 100 µm (Color figure available online).

In preparations double stained for p-tau and ferritin it is evident that NFD of grid cells is not accompanied by strong microglial ferritin expression (Figure 6a, b), unlike in many other instances. The microglia that express ferritin weakly often are dystrophic, as indicated by fragmented or atrophied processes (Figure 6b-d). TUNEL staining performed in selected cases (see Methods) reveals an exceptionally large number of TUNEL-positive microglia within the same region where grid cells are degenerating. TUNEL-positive microglia have an appearance similar to homeostatic microglia in that they have ramified processes (Figure 6e), which is expected as we have previously reported such cytoplasmic TUNEL staining of microglia.^35^ Occasionally, TUNEL-positive microglia show fragmentation of cytoplasmic extensions (Figure 6f). Cytoplasmic staining with TUNEL in ostensibly homeostatic microglia is likely due to the fact TUNEL labelling detects early-stage apoptosis, i.e., before nuclear fragmentation occurs.

## Discussion

The current study presents histopathological findings from humans that support a hypothesis of LOAD pathogenesis where NFD occurs because of elevated brain iron content and accompanying microglial senescence and exhaustion.^11,20^ With aging, there are increasingly high levels of iron in the brain that drive oxidative damage and microglial senescent degeneration, as well as ferroptosis of neurons. These damaging effects of iron dyshomeostasis are being counteracted by iron containment mechanisms that consist primarily of microglial ferritin expression and secondarily of amyloid deposition. While both containment mechanisms are active in all brain regions examined, they appear to be most effective in the more highly evolved isocortices. However, the effectiveness of neuroprotective iron sequestration by ferritin-positive microglia is limited as prolonged iron storage over time contributes to oxidative cell damage evident as microglial dystrophy,^25,38^ and possibly to microglial ferroptosis.^26,39–42^ We also report occurrence of apoptotic death of microglia *in situ*, as have others,^34,43,44^ yet we report it to be colocalized with degeneration of grid neurons, supporting the hypothesis that damage or death of microglia is detrimental for neuronal survival and promotes NFD due to compromised neuroprotection. While causes of microglial apoptosis *in situ* remain speculative, coexistence and colocalization of TUNEL-positive microglia with grid cell atrophic NFD suggest an interconnection, und underscore the importance of healthy microglia for neuronal survival.

Previously, we identified three potential iron containment mechanisms, i.e., microglial ferritin expression for intracellular sequestration in live cells, Aβ deposition for extracellular chelation, and NFT formation for terminal storage in dead neurons.^20^ In the current study, two of these were investigated within the context of how they may relate to the intensity of NFD. The fact that microglial ferritin expression occurred in all 34 individuals of our study suggests that it represents an important cellular mechanism for regulation of cerebral iron content. Deposition of Aβ seems to be a secondary mechanism that becomes deployed later in life, as evidenced by the fact that only some individuals in our cohort show Aβ deposits. We have shown in a prior study employing a larger cohort of non-demented individuals that Aβ deposition in the entorhinal cortex and hippocampus did not occur in subjects younger than age 61^33^ suggesting that Aβ production/deposition represents a final attempt of damming high iron levels in the aging brain when microglial iron sequestration becomes exhausted. Formation of neuritic plaques involves both accumulation of iron-laden ferritin-positive microglia and targeted deposition of Aβ at sites of neuronal ferroptosis, and thus *both* iron containment mechanisms are deployed during neuronal ferroptosis.^11^ Clusters of ferritin + microglia are an integral component of neuritic plaques,^45,46^ which indicates that neuritic plaques are sites where extraordinarily high levels of free iron are present^47^ which requires both iron sequestration in microglia as well as iron chelation *via* Aβ. Neuritic plaques are terminal lesions displaying maximal neuronal pathology, but also maximal deployment of iron containment mechanisms, and it seems clear that with aging the deployment of iron containment mechanisms increases in parallel with the neurodegenerative process. Containment of free iron may also be reflected by presence of vascular Aβ and ferritin. Our results show both vascular Aβ and ferritin + perivascular cells in neocortical regions. Perivascular cells constitute a subset of macrophages located within the perivascular spaces. Presence of vascular Aβ and ferritin + perivascular cells suggest high levels of free iron near the affected vessels. It could signify increased influx of iron from blood possibly due to a compromised blood-brain barrier (BBB).^48^ The fact that ferritin-positive microglia are found in all 34 subjects ranging from 54 to 93 years of age suggests that BBB damage and associated iron leakage occurs earlier in some subjects than in others. This is in line with the fact that, despite large age differences all subjects were at Braak stages II-III. It supports the notion that vascular health and BBB integrity are critically important for development of neurodegeneration. The same idea may also apply to development of chronic traumatic encephalopathy, a well-known tauopathy with environmental etiology.^49^ A recent study reports increased perivascular glial reactivity in chronic traumatic encephalopathy as a possible compensatory response to iron-induced oxidative stress.^50^

Our post-mortem work focused on lesion development during preclinical LOAD in non-demented human subjects has led us to postulate and corroborate the microglial dysfunction hypothesis, and present it as an alternative to the better known amyloid cascade hypothesis.^51^ One key finding in support of microglial dysfunction includes the identification of dystrophic microglia in the aging human brain.^24^ A key finding against the amyloid theory is the observation that amyloid plaques and NFTs are only rarely colocalized.^11^ At the same time, we noted that Aβ deposition occurs very precisely on neurons that have undergone droplet degeneration (ferroptosis) causing us to postulate a protective rather than a toxic role for Aβ.^12^ In the current study, we substantiate the microglial dysfunction theory but also the idea that iron dyshomeostasis is critical for triggering NFD in sporadic AD. The idea of iron dyshomeostasis fits well with observations that link it to classical AD lesions: amyloid deposits, neuritic plaques and NFTs are rich in iron,^12,13,47,52^ microglia are iron-laden,^11,53^ neuritic plaques originate from ferroptotic neurons.^20^ The idea is now being expanded to highlight potential relevance of iron containment mechanism. We show here that investigating deployment of iron containment mechanisms in the context of NFD offers improved understanding of iron's role in AD pathology. We show data suggesting that iron containment is less effective in allocortex than in isocortex in terms of reducing neuronal susceptibility to tau pathology. This may have to do with the simpler allocortical structure, and/or its ontogenetic development, i.e., older structures are more vulnerable to high iron, which is supported by the fact that older structures in the brainstem (locus coeruleus) are affected before cortical neurons.^2^ Thus, differential neuronal vulnerability between allo-and isocortex may occur because of more effective neuroprotection *via* ferritin-expressing microglia and Aβ deposition in isocortex. Better neuroprotection may be possible because isocortical neurons are “easier” to protect due to lower metabolic or trophic demands than allocortical neurons, which is supported by the current observations showing that ferritin-expressing microglia are present to near-equal extents in both allo-and isocortex. Microglia expressing ferritin early on during preclinical disease are healthy cells that provide neuroprotection through iron sequestration. However, during more advanced disease and dementia, ferritin + microglia begin to show signs of damage and death, which may be due to exhaustion and ultimately the demise of these damaged cells *via* apoptosis or ferroptosis.^34,54^ A possible example of insufficient microglial neuroprotection may be seen in individual 8 (Table 1), where NFD of grid neurons coincides not only with microglial apoptosis, but also with weak ferritin expression and microglial dystrophy, as well as an absence of Aβ deposits. Case 8 appears to have been somebody particularly susceptible to development of AD pathology, not only because of insufficient neuroprotection but also because case 8 is the only individual in our cohort who revealed NFD of grid cells, which has not previously been shown to occur in non-demented humans. The constellation of age, microglial pathology and uncommon NFD dramatically emphasizes the interdependence of neurons and microglia in this particular case. While most neurons undergoing atrophic NFD were grid cells located in the medial entorhinal cortex, other stellate neurons in the dentate hilus (possibly place cells) also revealed the same kind of atrophic NFD. The colocalization of grid neuron degeneration and microglial apoptosis could mean that neurons undergoing atrophic NFD are particularly sensitive to microglial death and associated loss of neurotrophic support.

## References

[1] Braak H Braak E . Neuropathological stageing of Alzheimer-related changes. Acta Neuropathol 1991; 82: 239–259. 1759558 10.1007/BF00308809

[2] Braak H Del Tredici K . The pathological process underlying Alzheimer’s disease in individuals under thirty. Acta Neuropathol 2011; 121: 171–181. 21170538 10.1007/s00401-010-0789-4

[3] Braak H Thal DR Ghebremedhin E , et al. Stages of the pathologic process in Alzheimer disease: age categories from 1 to 100 years. J Neuropathol Exp Neurol 2011; 70: 960–969. 22002422 10.1097/NEN.0b013e318232a379

[4] Braak H Del Trecidi K . Neuroanatomy and pathology of sporadic Alzheimer’s disease. Adv Anat Embryol Cell Biol 2015; 215: 1–162. 25920101

[5] Hardy J Selkoe DJ . The amyloid hypothesis of Alzheimer’s disease: progress and problems on the road to therapeutics. Science 2002; 297: 353–356. 12130773 10.1126/science.1072994

[6] Kayed R Head E Thompson JL , et al. Common structure of soluble amyloid oligomers implies common mechanism of pathogenesis. Science 2003; 300: 486–489. 12702875 10.1126/science.1079469

[7] Glabe CG . Common mechanisms of amyloid oligomer pathogenesis in degenerative disease. Neurobiol Aging 2006; 27: 570–575. 16481071 10.1016/j.neurobiolaging.2005.04.017

[8] Crary JF Trojanowski JQ Schneider JA , et al. Primary age-related tauopathy (PART): a common pathology associated with human aging. Acta Neuropathol 2014; 128: 755–766. 25348064 10.1007/s00401-014-1349-0 PMC4257842

[9] Braak H Del Tredici K . Are cases with tau pathology occurring in the absence of Abeta deposits part of the AD-related pathological process? Acta Neuropathol 2014; 128: 767–772. 25359108 10.1007/s00401-014-1356-1

[10] Duyckaerts C Braak H Brion JP , et al. PART Is part of Alzheimer disease. Acta Neuropathol 2015; 129: 749–756. 25628035 10.1007/s00401-015-1390-7 PMC4405349

[11] Streit WJ Rotter J Winter K , et al. Droplet degeneration of hippocampal and cortical neurons signifies the beginning of neuritic plaque formation. J Alzheimers Dis 2022; 85: 1701–1720. 34958037 10.3233/JAD-215334

[12] Castellani RJ Lee HG Siedlak SL , et al. Reexamining Alzheimer’s disease: evidence for a protective role for amyloid-beta protein precursor and amyloid-beta. J Alzheimers Dis 2009; 18: 447–452. 19584435 10.3233/JAD-2009-1151 PMC2907530

[13] Smith MA Harris PL Sayre LM , et al. Iron accumulation in Alzheimer disease is a source of redox-generated free radicals. Proc Natl Acad Sci U S A 1997; 94: 9866–9868. 9275217 10.1073/pnas.94.18.9866 PMC23283

[14] Peters DG Connor JR Meadowcroft MD . The relationship between iron dyshomeostasis and amyloidogenesis in Alzheimer’s disease: two sides of the same coin. Neurobiol Dis 2015; 81: 49–65. 26303889 10.1016/j.nbd.2015.08.007 PMC4672943

[15] Collingwood JF Mikhaylova A Davidson M , et al. In situ characterization and mapping of iron compounds in Alzheimer’s disease tissue. J Alzheimers Dis 2005; 7: 267–272. 16131727 10.3233/jad-2005-7401

[16] Castellani RJ Moreira PI Perry G , et al. The role of iron as a mediator of oxidative stress in Alzheimer disease. Biofactors 2012; 38: 133–138. 22447715 10.1002/biof.1010

[17] Ayton S Portbury S Kalinowski P , et al. Regional brain iron associated with deterioration in Alzheimer’s disease: a large cohort study and theoretical significance. Alzheimers Dement 2021; 17: 1244–1256. 33491917 10.1002/alz.12282 PMC9701539

[18] Zecca L Youdim MB Riederer P , et al. Iron, brain ageing and neurodegenerative disorders. Nat Rev Neurosci 2004; 5: 863–873. 15496864 10.1038/nrn1537

[19] Thorwald MA Godoy-Lugo JA Garcia G , et al. Iron-associated lipid peroxidation in Alzheimer’s disease is increased in lipid rafts with decreased ferroptosis suppressors, tested by chelation in mice. Alzheimers Dement 2025; 21: e14541. 10.1002/alz.14541 PMC11775463 39876821

[20] Streit WJ Phan L Bechmann I . Ferroptosis and pathogenesis of neuritic plaques in Alzheimer disease. Pharmacol Rev 2025; 77: 100005. 39952690 10.1124/pharmrev.123.000823

[21] Braak H Rub U Schultz C , et al. Vulnerability of cortical neurons to Alzheimer’s and Parkinson’s diseases. J Alzheimers Dis 2006; 9: 35–44. 10.3233/jad-2006-9s305 16914843

[22] Zecca L Gallorini M Schunemann V , et al. Iron, neuromelanin and ferritin content in the substantia nigra of normal subjects at different ages: consequences for iron storage and neurodegenerative processes. J Neurochem 2001; 76: 1766–1773. 11259494 10.1046/j.1471-4159.2001.00186.x

[23] Ward RJ Zucca FA Duyn JH , et al. The role of iron in brain ageing and neurodegenerative disorders. Lancet Neurol 2014; 13: 1045–1060. 25231526 10.1016/S1474-4422(14)70117-6 PMC5672917

[24] Streit WJ Braak H Xue QS , et al. Dystrophic (senescent) rather than activated microglial cells are associated with tau pathology and likely precede neurodegeneration in Alzheimer’s disease. Acta Neuropathol 2009; 118: 475–485. 19513731 10.1007/s00401-009-0556-6 PMC2737117

[25] Lopes KO Sparks DL Streit WJ . Microglial dystrophy in the aged and Alzheimer’s disease brain is associated with ferritin immunoreactivity. Glia 2008; 56: 1048–1060. 18442088 10.1002/glia.20678

[26] Adeniyi PA Gong X MacGregor E , et al. Ferroptosis of microglia in aging human white matter injury. Ann Neurol 2023; 94: 1048–1066. 37605362 10.1002/ana.26770 PMC10840747

[27] Fendrick SE Xue QS Streit WJ . Formation of multinucleated giant cells and microglial degeneration in rats expressing a mutant Cu/Zn superoxide dismutase gene. J Neuroinflammation 2007; 4: 9. 17328801 10.1186/1742-2094-4-9 PMC1808448

[28] Streit WJ . Microglia and Alzheimer’s disease pathogenesis. J Neurosci Res 2004; 77: 1–8. 15197750 10.1002/jnr.20093

[29] Streit WJ Xue QS . Alzheimer’s disease, neuroprotection, and CNS immunosenescence. Front Pharmacol 2012; 3: 138. 22822399 10.3389/fphar.2012.00138 PMC3398410

[30] Swanson MEV Scotter EL Smyth LCD , et al. Identification of a dysfunctional microglial population in human Alzheimer’s disease cortex using novel single-cell histology image analysis. Acta Neuropathol Commun 2020; 8: 170. 33081847 10.1186/s40478-020-01047-9 PMC7576851

[31] Braak H Alafuzoff I Arzberger T , et al. Staging of Alzheimer disease-associated neurofibrillary pathology using paraffin sections and immunocytochemistry. Acta Neuropathol 2006; 112: 389–404. 16906426 10.1007/s00401-006-0127-z PMC3906709

[32] Thal DR Rub U Orantes M , et al. Phases of A beta-deposition in the human brain and its relevance for the development of AD. Neurology 2002; 58: 1791–1800. 12084879 10.1212/wnl.58.12.1791

[33] Streit WJ Braak H Del Tredici K , et al. Microglial activation occurs late during preclinical Alzheimer’s disease. Glia 2018; 66: 2550–2562. 30417428 10.1002/glia.23510

[34] Lassmann H Bancher C Breitschopf H , et al. Cell death in Alzheimer’s disease evaluated by DNA fragmentation in situ. Acta Neuropathol (Berl) 1995; 89: 35–41. 7709729 10.1007/BF00294257

[35] Conde JR Streit WJ . Effect of aging on the microglial response to peripheral nerve injury. Neurobiol Aging 2006; 27: 1451–1461. 16159684 10.1016/j.neurobiolaging.2005.07.012

[36] Simmons DA Casale M Alcon B , et al. Ferritin accumulation in dystrophic microglia is an early event in the development of Huntington’s disease. Glia 2007; 55: 1074–1084. 17551926 10.1002/glia.20526

[37] Hafting T Fyhn M Molden S , et al. Microstructure of a spatial map in the entorhinal cortex. Nature 2005; 436: 801–806. 15965463 10.1038/nature03721

[38] Neumann P Lenz DE Streit WJ , et al. Is microglial dystrophy a form of cellular senescence? An analysis of senescence markers in the aged human brain. Glia 2023; 71: 377–390. 36286188 10.1002/glia.24282

[39] Cai Z Wang S Cao S , et al. Loss of ATG7 in microglia impairs UPR, triggers ferroptosis, and weakens amyloid pathology control. J Exp Med 2025; 222: e20230173. 10.1084/jem.20230173 PMC11823820 39945772

[40] Sun GG Wang C Mazzarino RC , et al. Microglial APOE3 Christchurch protects neurons from Tau pathology in a human iPSC-based model of Alzheimer’s disease. Cell Rep 2024; 43: 114982. 39612244 10.1016/j.celrep.2024.114982 PMC11753789

[41] Ryan SK Zelic M Han Y , et al. Microglia ferroptosis is regulated by SEC24B and contributes to neurodegeneration. Nat Neurosci 2023; 26: 12–26. 36536241 10.1038/s41593-022-01221-3 PMC9829540

[42] Kenkhuis B Bush AI Ayton S . How iron can drive neurodegeneration. Trends Neurosci 2023; 46: 333–335. 36842947 10.1016/j.tins.2023.02.003

[43] Jellinger KA Stadelmann CH . The enigma of cell death in neurodegenerative disorders. J Neural Transm Suppl 2000: 21–36. 11205141 10.1007/978-3-7091-6301-6_2

[44] Jellinger KA Stadelmann C . Problems of cell death in neurodegeneration and Alzheimer’s disease. J Alzheimers Dis 2001; 3: 31–40. 12214070 10.3233/jad-2001-3106

[45] Grundke-Iqbal I Fleming J Tung YC , et al. Ferritin is a component of the neuritic (senile) plaque in Alzheimer dementia. Acta Neuropathol 1990; 81: 105–110. 2082650 10.1007/BF00334497

[46] Kaneko Y Kitamoto T Tateishi J , et al. Ferritin immunohistochemistry as a marker for microglia. Acta Neuropathol 1989; 79: 129–136. 2596262 10.1007/BF00294369

[47] Lovell MA Robertson JD Teesdale WJ , et al. Copper, iron and zinc in Alzheimer’s disease senile plaques. J Neurol Sci 1998; 158: 47–52. 9667777 10.1016/s0022-510x(98)00092-6

[48] Montagne A Barnes SR Sweeney MD , et al. Blood-brain barrier breakdown in the aging human hippocampus. Neuron 2015; 85: 296–302. 25611508 10.1016/j.neuron.2014.12.032 PMC4350773

[49] McKee AC Cantu RC Nowinski CJ , et al. Chronic traumatic encephalopathy in athletes: progressive tauopathy after repetitive head injury. J Neuropathol Exp Neurol 2009; 68: 709–735. 19535999 10.1097/NEN.0b013e3181a9d503 PMC2945234

[50] Osterman C Hamlin D Suter CM , et al. Perivascular glial reactivity is a feature of phosphorylated tau lesions in chronic traumatic encephalopathy. Acta Neuropathol 2025; 149: 16. 39921702 10.1007/s00401-025-02854-x PMC11807024

[51] Streit WJ Khoshbouei H Bechmann I . The role of microglia in sporadic Alzheimer’s disease. J Alzheimers Dis 2021; 79: 961–968. 33361603 10.3233/JAD-201248

[52] Bishop GM Robinson SR . The amyloid paradox: amyloid-beta-metal complexes can be neurotoxic and neuroprotective. Brain Pathol 2004; 14: 448–452. 15605992 10.1111/j.1750-3639.2004.tb00089.x PMC8095825

[53] Kenkhuis B Somarakis A de Haan L , et al. Iron loading is a prominent feature of activated microglia in Alzheimer’s disease patients. Acta Neuropathol Commun 2021; 9: 27. 33597025 10.1186/s40478-021-01126-5 PMC7887813

[54] Streit WJ Xue QS Tischer J , et al. Microglial pathology. Acta Neuropathol Commun 2014; 2: 142. 25257319 10.1186/s40478-014-0142-6 PMC4180960

